# SG-APSIC1095: Acquisition rate of carbapenemase-producing organisms (CPOs) among hospital contacts of CPO patients: An interim subgroup analysis of a cohort study

**DOI:** 10.1017/ash.2023.70

**Published:** 2023-03-16

**Authors:** Sharifah Farhanah Syed Husen, Kyaw Zaw Linn, Clara Chong Hui Ong, Xiaowei Huan, Liang Hui Loo, Ismail Bin Sazali, Shawn Vasoo, Meow Ling Foo, Say Tat Ooi, Thean Yen Tan, Nares Smitasin, Moi Lin Ling, Paul Anantharajah Tambyah, Seow Yen Tan, Oon Tek Ng, Kalisvar Marimuthu

**Affiliations:** National Centre for Infectious Diseases, Singapore; National Public Health & Epidemiology Unit, National Centre for Infectious Diseases, Singapore; Infectious Disease Research and Training Office, National Centre for Infectious Diseases, Singapore; Infectious Disease Research and Training Office, National Centre for Infectious Diseases, Singapore; Infectious Disease Research and Training Office, National Centre for Infectious Diseases, Singapore; Infection Prevention & Epidemiology, Singapore General Hospital, Singapore; Infectious Diseases, Tan Tock Seng Hospital, Singapore; Infection Control, Khoo Teck Puat Hospital, Singapore; General Medicine, Khoo Teck Puat Hospital, Singapore; Lab Medicine, Changi General Hospital, Singapore; Infectious Diseases, National University Hospital, Singapore; Infection Prevention & Epidemiology, Singapore General Hospital, Singapore; Infectious Diseases, National University Hospital, Singapore; Infectious Diseases, Changi General Hospital, Singapore; Infectious Diseases, Tan Tock Seng Hospital, Singapore; Infectious Diseases, Tan Tock Seng Hospital, Singapore

## Abstract

**Objectives:** The increase in carbapenemase-producing organism (CPO) transmission among hospitalized patients is a growing concern. Studies investigating the transmission of CPO to epidemiologically linked contacts are scarce. We conducted an interim subgroup analysis of the ongoing multicenter household transmission of CPO in Singapore (CaPES-C) study to identify the acquisition rate of CPO among epidemiologically linked contacts of hospitalized CPO patients. **Methods:** This multicenter prospective cohort study was conducted between January and December 2021. We recruited CPO-positive patients and their epidemiologically linked contacts. Stool samples were collected from the patients at baseline, day 3, day 7, and at weeks 2, 3, 4, 5, 6, 12, 24, 36, and 48. Additionally, a sample was collected at the time of discharge from the hospital. Xpert Carba-R test was used to detect CPO genotypes in the stool samples. In this interim analysis, we calculated the acquisition rate of CPO among the epidemiologically linked hospital contacts of CPO positive patients using Stata version 15 software. **Results:** We recruited 22 (56.4%) CPO-positive index patients [*bla*NDM, n = 7 (31.8%); *bla*IMP, n = 3 (13.6%); *bla*OXA-48, n = 10 (45.5%), others, n = 2 (9.1%)] and 14 (35.9%) epidemiologically linked hospital contacts. The median age of CPO-positive patients was 72.5 years (IQR, 62–82) and 15 (68.2%) were female. The median age for the epidemiologically linked contacts was 82.5 years (IQR, 70–85) and 4 (28.6%) were female. After 1,082 patient days, 2 (14.3%) epidemiologically linked contacts tested positive for CPO giving an acquisition rate of 1.85 per 1,000 patient days (95% CI, 0.46 – 7.39). One of these participants acquired a concordant genotype (*bla*OXA-48) at day 7 and the other acquired a discordant genotype (CPO positive index, *bla*IMP; epidemiologically linked contact, *bla*NDM) at week 12 of follow-up. **Conclusions:** This small interim analysis revealed a high conversion rate among epidemiologically linked hospital contacts. A larger study is needed to understand the influence of genotypes, hospital environment, and human behavior on the transmission of CPO in hospitals.

